# The budget impact of procalcitonin-guided antibiotic stewardship compared to standard of care for patients with suspected sepsis admitted to the intensive care unit in Belgium

**DOI:** 10.1371/journal.pone.0293544

**Published:** 2023-10-30

**Authors:** Victoria Madeleine Garnfeldt, Jean-Louis Vincent, Damien Gruson, Osvaldo Ulises Garay, Stefaan Vansieleghem, Leonardo Iniguez, Alexander Lefevre

**Affiliations:** 1 Department of Health Science and Technology, Aalborg University, Gistrup, Denmark; 2 Government, Access & Patient Affairs, Roche Diagnostics Belgium, Brussels, Belgium; 3 Department of Intensive Care, Erasme, University Hospital, Université Libre de Bruxelles, Brussels, Belgium; 4 Department of Clinical Biochemistry, Cliniques Universitaires St-Luc, Université Catholique de Louvain, Brussels, Belgium; 5 Access Evidence, Roche Diagnostics International Ltd., Rotkreuz, Switzerland; 6 Hebias, Ronse, Belgium; 7 Marketing and Medical Excellence, Roche Diagnostics Belgium, Brussels, Belgium; Jinan University First Affiliated Hospital, CHINA

## Abstract

In Belgium, antibiotic resistance leads to approximately 530 deaths with a €24 million financial burden annually. This study estimated the impact of procalcitonin-guided antibiotic stewardship programs to reduce antibiotic consumption versus standard of care in patients with suspected sepsis. A decision analytic tree modelled health and budget outcomes of procalcitonin-guided antibiotic stewardship programs for patients admitted to the intensive care unit (ICU). A literature search, a survey with local clinical experts, and national database searches were conducted to obtain model input parameters. The main outcomes were total budget impact per patient, reduction in number of antibiotic resistance cases, and cost per antibiotic day avoided. To evaluate the impact of parameter uncertainty on the source data, a deterministic sensitivity analysis was performed. A scenario analysis was conducted to investigate budget impact when including parameters for reduction in length of ICU stay and mechanical ventilation duration, in addition to base-case parameters. Based on model predictions, procalcitonin-guided antibiotic stewardship programs could reduce the number of antibiotic days by 66,868, resulting in €1.98 million savings towards antibiotic treatment in current clinical practice. Antibiotic resistance cases could decrease by 7.7% (6.1% vs 9.2%) in the procalcitonin-guided setting compared with standard of care. The base-case budget impact suggests an investment of €1.90 per patient. The sensitivity analysis showed uncertainty, as the main drivers can alter potential cost savings. The scenario analysis indicated a saving of €1,405 per patient, with a reduction of 1.5 days in the ICU (14.8 days vs 12.8 days), and a reduction of 22.7% (18.1–27.2%) in mechanical ventilation duration. The associated sensitivity analysis was shown to be robust in all parameters. Procalcitonin-guided antibiotic stewardship programs are associated with clinical benefits that positively influence antimicrobial resistance in Belgium. A small investment per patient to implement procalcitonin testing may lead to considerable cost savings.

## Introduction

Ever since the antimicrobial properties of chemicals secreted by the *Penicillium* moulds were discovered as the first antimicrobial agents in 1928, antibiotics have enabled the treatment of formerly fatal diseases [1]. Inappropriate use of antibiotics is increasing the incidence of antimicrobial resistance (AMR), thus reducing their effectiveness, and posing a global health issue [2]. In Europe, there are approximately 33,000 deaths annually due to AMR infections, and it is estimated that 530 of these occur in Belgium [3]. The economic burden of AMR in Belgium alone is estimated to be €24 million (M) per year and is predicted to increase to €787M per year by 2050 [3, 4].

Antibiotic treatment can be crucial in treating fatal diseases, such as sepsis [5]. Sepsis is defined as life-threatening organ dysfunction caused by a dysregulated response to infection by the host and is one of the leading causes of mortality worldwide [6]. Sepsis is a major cause of morbidity and mortality in the intensive care unit (ICU) [6]. In a global audit (84 countries) comprising of 10,069 patients admitted to the ICU, sepsis was identified in 29.5%; of these patients, 18.0% were already admitted to the ICU [7]. Across the 84 countries, the average sepsis mortality rate was 25.8% [7]. The severity of the complication, and international guidelines recommending urgent intervention for sepsis management, leave physicians with a short time frame to initiate treatment. According to guidelines, antibiotic administration is recommended within 1–3 hours, which may cause physicians to disregard diagnostic examinations to meet the deadline [5, 8]. Multiple studies from the Netherlands and France have shown that up to 43% of patients with suspected sepsis are unnecessarily treated with antibiotics, thus contributing to AMR [9–11].

To address the globally increasing incidence of AMR, antibiotic stewardship programs (ASPs) have been developed as part of country-specific action plans, derived from and in line with the World Health Organization’s global action plan [12, 13]. ASPs aim to preserve the effectiveness of antibiotics and can be defined as actions of a healthcare institution to reform and improve the use of antibiotics in patients (both administration and discontinuation), improve health outcomes, reduce AMR, and secure cost-effective treatments [12]. ASPs are of pan-global responsibility, as the pathogenic nature of microbes and AMR prevalence is not restricted to frontiers or country borders. In Belgium, the most recent action plan is for 2020–2024, and focuses on AMR in humans, animals, plants, and the environment [4].

The global overuse of antibiotics has led to the investigation of potential biomarkers to help distinguish between bacterial and viral infections and to improve ASPs in hospitals [14, 15]. One biomarker, procalcitonin (PCT), has been found to be superior in distinguishing between infectious etiologies compared with other biomarkers [14]. The PCT peptide is a precursor of the hormone calcitonin and is only present at low and undetectable serum levels in healthy individuals. In response to bacterial infections, circulating PCT increases to detectable levels after 3–4 hours and may undergo a 1,000-fold increase. Within 14–24 hours, the serum levels of PCT are at their highest. The half-life of PCT is between 20–35 hours; thus, serum levels decrease by approximately 50% per day when the bacterial infection is treated correctly [16, 17]. Several assays for PCT are available, allowing a rapid measurement in clinical laboratories through automated immunoassays or close to patients through point of care testing. Recently, studies have proven the effectiveness of using PCT as a tool for antibiotic stewardship, providing insights on initiation and discontinuation of antibiotic treatment in patients with suspected sepsis [18, 19]. PCT-guided ASPs may be invaluable in providing insights to physicians to allow for the appropriate administration of treatment within the recommended 1–3 hours in patients with suspected sepsis, which is crucial for their recovery [5, 8]. Additionally, PCT-guided ASPs may help physicians to decide on when to discontinue antibiotic use following clinical stabilization, preventing patient harm and resistance development and also reducing antibiotic therapy duration, which would result in cost savings [20].

When new interventions are presented, it is important to outline not only the effectiveness of the new intervention, but also the budgetary impact. This is done through health economic evaluations to inform decision-makers, which will ultimately benefit the healthcare system [19]. Health economic evaluations and cost analyses in patients with lower respiratory tract infections, or suspected sepsis in the ICU have been done for PCT-guided antibiotic stewardship in various countries, such as Argentina, the Netherlands, and the United States (US) [20–22]. To our knowledge, there have not been any budgetary analyses carried out in Belgium comprising patients with suspected sepsis in the ICU; thus, the aim of this study is to determine the budget impact of PCT-guided antibiotic stewardship in patients with suspected sepsis in the Belgian healthcare system, specifically those admitted to the ICU.

## Materials and methods

**Fig 1:**
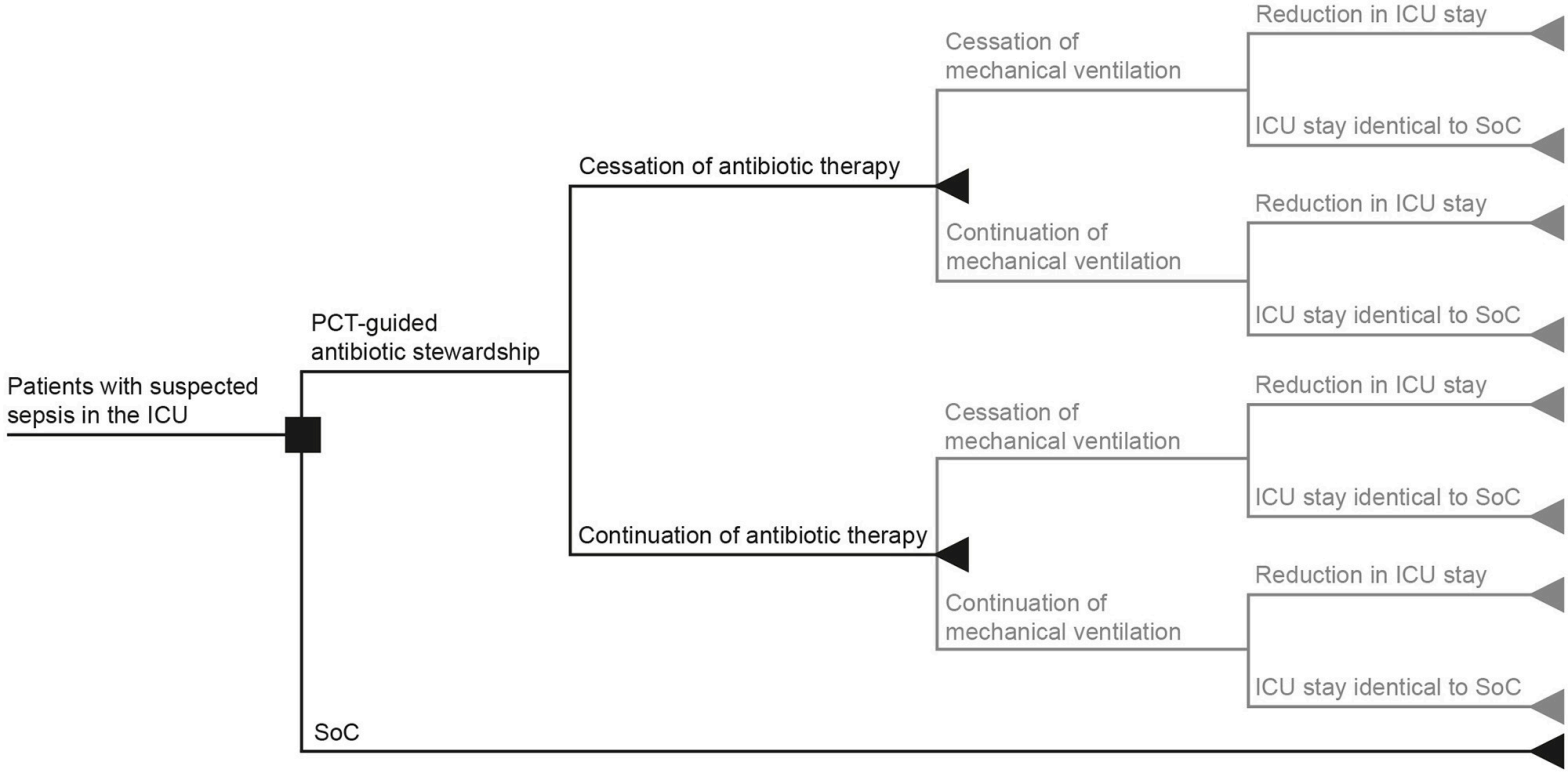
Decision tree adapted for Belgium. Decision tree for patients with suspected sepsis. The base-case analysis is presented in black, and the scenario analysis is represented by the grey branches.

This budget impact analysis simulates the cost and clinical effectiveness of PCT-guided antibiotic stewardship versus standard of care (SoC) for all patients with suspected sepsis in the ICU. The analysis is based on previously published decision trees [21, 22], adapted to the Belgian healthcare system (Fig 1), where PCT tests are not reimbursed, thus not consistently used. For the reporting of results of economic assessments, the study followed the Belgian guidelines for economic evaluations and budget impact analyses [23], the International Society for Pharmacoeconomics and Outcomes budget impact analysis principles of good practice [24], and the Consolidated Health Economic Evaluation Reporting Standards statement [25]. The analysis was conducted from the perspective of the healthcare system over one year; thus, discount rates were not relevant. In alignment with Belgian guidelines [23], all costs prior to 2023 were inflated using the most recent health index figures (January 2023) from Statbel, and expressed in euros [26].

The primary outcome was the budget impact per year on the Belgian healthcare system for both PCT-guided antibiotic stewardship and SoC. Secondary outcomes included antibiotic therapy duration and a reduction in the number of expected AMR infections per year.

A literature review, structured as a PICO (population, intervention, comparison, outcome) search, was carried out in three online medical databases (PubMed, Embase, and The Cochrane Central Register of Controlled Trials) to investigate the effectiveness of PCT versus SoC. The search strategy included articles written in English from 2017–2022. In addition, an expert survey was designed to obtain the Belgium-specific model input of PCT-guided antibiotic stewardship versus SoC, which could not be found in literature or elsewhere. The survey was conducted by each expert individually and collated to form a single conclusion.

### Model parameters

All base-case parameters with minimum and maximum values used for sensitivity analyses are presented in Table 1. A conservative assumption was made that for each parameter, a ±20% range of variability was applied to enable ranking of parameters in the sensitivity analyses. Furthermore, if no patient-specific costs were indicated, the patient out-of-pocket cost was assumed to be 10% of All Patients Refined Diagnosis Related Groups (APR-DRG) costs, available from Minimum Hospital Data (Résumé Hospitalier Minimum) 2016–2020, de dato 09 03 2022; Data and Policy Information Service, FPS Public Health, Food Chain Safety and Environment, Belgium (henceforth referred to as FPS Public Health) [27, 28].

**Table 1:** Base-case parameters used for sepsis sensitivity analysis.

Parameter	Base (range)	Sources
*Patients with sepsis in the ICU*		
**Epidemiology**		
Sepsis cases, per year	35,516 (28,413–42,619)	Federal Public Service (FPS) Public Health [29]
AMR in population, %	9.4 (7.5–11.1)	FPS Public Health [29]
**Resource use**		
SoC		
Time on a regular ward, days	5.1 (4.1–6.1)	Assumption, APR-DRG [27], FPS public health [29]
LOS in the ICU, days	10.6 (8.4–12.7)	Assumption, APR-DRG [27], FPS public health [29]
Antibiotic therapy duration, days	8.5 (6.8–10.2)	Expert opinion, APR-DRG [28]
Patients on mechanical ventilation, %	57.5 (46.0–69.0)	Expert opinion
Mechanical ventilation duration, days	6.3 (5.0–7.6)	Expert opinion
Length of stay on regular ward due to AMR, days	7.0 (5.6–8.4)	Expert opinion
PCT		
PCT test per patient, n	3 (2.4–3.6)	Expert opinion
**PCT antibiotic stewardship effectiveness**		
Reduction of antibiotic therapy duration, %	22.5 (18.0–27.0)	Study [19]
Reduction of AMR in population, %	7.7 (6.1–9.2)	Study [21, 22, 30]
*Unit costs*, *€*		
Cost of stay on regular ward, per day	587.60 (470.10–705.10)	Assumption, APR-DRG [27]
Cost of stay in the ICU, per day	881.40 (705.10–1,057.60)	Assumption, APR-DRG [27]
Cost of antibiotic therapy, per day	29.20 (23.30–35.00)	Assumption, APR-DRG [28]
Cost of mechanical ventilation, per day	144.80 (115.80–173.80)	NomenSoft [31]
Cost of PCT test, per patient	29.10 (23.30–34.90)	Study [32]

### Epidemiology

The annual cases of patients with sepsis in Belgium were obtained by request from FPS Public Health and based on the sum of four International Classification of Diseases-10 codes: A40, A41, O85, and P36 [29]. The number of patients with sepsis was estimated based on the average sepsis prevalence from 2016–2020 and extrapolated to 2023 using the annual growth rate of 1.49% in Belgium [26, 33]. The proportion of patients with sepsis developing AMR in the ICU in 2020 was 9.4% (range: 7.5–11.2) and was obtained from FPS Public Health [29].

### Resource use

Data on hospital length of stay (LOS) for patients with sepsis were obtained from FPS Public Health [29]. Using data from 2020, the mean LOS was estimated, as a weighted average based on the severity of disease, to be 15.3 days (range: 12.3–18.4) [27]. The proportion of the LOS allocated to the ICU was 67.4% (range: 53.9–80.9), based on the study from Mewes et al. [22]. Thus, in consensus with experts, the LOS on a regular ward was 5.0 days (range: 4.0–6.0) and the LOS in the ICU was 10.3 days (range: 8.3–12.4). According to experts, an average of 57.5% (range: 46.0–69.0) of patients with sepsis received mechanical ventilation for a duration of 6.3 days (range: 5.0–7.6). The additional LOS on a regular ward for patients who developed AMR was 7 days (range: 5.6–8.4), based on expert opinion. As PCT tests are not routinely used, the most optimal number of PCT tests was estimated to be 3 per patient (range: 2.4–3.6) based on the expert survey.

### Effectiveness of PCT antibiotic stewardship

This study was based on previously published studies that performed health economic evaluations for PCT-guided antibiotic stewardship in various countries, [21, 22] and was adapted to the Belgian healthcare system; thus, base-case and scenario analyses were conducted (Fig 1). The base-case analysis investigated the effectiveness of PCT based on antibiotic consumption and AMR cases. In parallel with the base-case analysis, a scenario analysis was performed to illustrate the potential effects of PCT-guided antibiotic stewardship, such as a reduction in LOS in the hospital, and mechanical ventilation duration.

The reduction in antibiotic therapy duration was estimated to be 22.5% (range: 18.0–27.0%) based on a study by Gutiérrez-Pizarraya et al. [19]. To calculate the reduction in antibiotic therapy duration, weighted averages of the total antibiotic duration in days were calculated from the PCT and SoC groups to obtain the difference in the total duration of antibiotic therapy between groups. The difference between groups was then converted into a percentage.

Several studies have estimated the percentage reduction in AMR due to PCT by using the proportion of AMR in the PCT and SoC settings, and the calculated reduction in antibiotic duration due to PCT-guided antibiotic stewardship https://www.zotero.org/google-docs/?Y6l7lB [21, 22, 30]. This study followed the methodologic approach of the previously published studies; the proportion of AMR in the PCT setting was calculated by multiplying the 3.2% reduction in AMR with the calculated percentage reduction in time on antibiotic therapy due to PCT and subtracting this from the proportion of AMR in the SoC setting. Then, the proportion of AMR in the PCT setting was divided by the proportion of AMR in SoC and subtracted from one to obtain the reduction in AMR due to PCT. The reduction in AMR due to PCT was 7.7% (range: 6.1–9.2%) [19, 22, 30].

### Unit cost estimates

The cost of 12.8 hospital days for sepsis was €11,168.80, based on the APR-DRG-720 (APR-DRG code for sepsis) [27]. Due to a difference in LOS between data sources (APR-DRG and FPS Public Health), the unit cost per day was estimated based on data from the APR-DRG alone. As the data did not differentiate between a regular ward and the ICU, or between government costs and patient costs, assumptions were made that the cost of ICU admission was 50% higher compared with admission to a regular ward, and that patient out-of-pocket co-payment was 10% of any APR-DRG cost. The cost of admission to the ICU was €881.40 (range: €705.10–1,057.60) per day and the cost of admission to a regular ward was €587.60 (range: €470.10–705.10) per day [27]. The average cost of antibiotic treatment per hospital stay was obtained using the APR-DRG-720 code for sepsis. The cost per day was calculated by dividing the total cost of antibiotic treatment by the treatment duration; thus, the cost of antibiotic treatment was €29.20 (range: €23.30–35.00) per day [28]. To estimate the cost of mechanical ventilation, the NomenSoft [31] database for reimbursed healthcare services was used. Two nomenclature codes were applied, 211363 and 211385, representing the cost of the first day and the cost of the following day(s) of mechanical ventilation [31]. Based on the two nomenclature codes and the mechanical ventilation duration, the estimated cost was €144.80 (range: €115.80–173.80) per day [31]. As PCT is not reimbursed in Belgium, there is no official data on assay cost. The price of one PCT assay, based on a Dutch study by Kip et al, was estimated to be €38.80 (range: €31.00–46.50); as it is assumed the government will cover 75% of this cost, the cost of each PCT assay was assumed to be €29.10 (range: €23.30–34.90) [32].

### Sensitivity analysis and scenario analysis

To evaluate the robustness of the results, a deterministic sensitivity analysis was performed. As the ±20% range of variability was applied, it was possible to rank the drivers of the results and summarize them in a top-down tornado diagram. The tornado diagram illustrates the univariate analysis where one parameter was altered to the extreme of its range of variability whilst all other parameters remained unchanged.

In parallel to the base-case analysis, a scenario analysis was performed, based on published literature, to illustrate the potential budget impact of a reduction in hospital LOS and mechanical ventilation duration (Table 2). A study by Andriolo et al, showed that PCT caused a 13.9% (range: 11.1–16.6%) reduction in LOS in the ICU; however, no reduction in LOS on a regular ward was indicated [18]. The reduction in LOS in the ICU was calculated similarly to the reduction in antibiotic therapy duration, using weighted averages of LOS in the ICU from the PCT and SoC groups to obtain the difference in total LOS in the ICU for each group. The difference between groups was then converted into a percentage.

**Table 2:** Scenario analysis for patients with sepsis in the ICU. Parameters altered from the base-case on effectiveness of PCT antibiotic stewardship.

Parameter	Base (range)	Sources
*Patients with sepsis in the ICU*		
**PCT antibiotic stewardship effectiveness, %**		
Reduction in LOS in the ICU	13.9 (11.1–16.6)	Study [18]
Reduction in mechanical ventilation duration	22.7 (18.1–27.2)	Study [22]

According to the study by Mewes et al. [22], the reduction in mechanical ventilation duration was calculated by multiplying the days on mechanical ventilation per 1,000 patient days by the reduction in LOS in the ICU. The calculations were based on a 10.2% reduction in days on mechanical ventilation per 1,000 patient days due to PCT, obtained from the study by Mewes et al. [22]. The calculated reduction in mechanical ventilation duration was 22.7% (range: 18.1–27.2%).

## Results

The results of the model-based analysis (Table 3) indicated that PCT as a tool for antibiotic stewardship can be associated with a reduction in antibiotic exposure by 66,868 days, resulting in cost savings of €1.98M towards antibiotic treatment per year. Furthermore, 256 AMR infections can be avoided when performing an average of three PCT tests per patient; however, the implementation of PCT-guided antibiotic stewardship for patients with suspected sepsis in Belgium would require a budget investment of €68,220 (a cost of €1.90 per patient).

**Table 3:** Expected results. Healthcare impact.

	SoC	PCT	Difference
**Healthcare impact, per year**			
Total antibiotic days for all patients, per year*	301,884	234,016	–66,868
Cases of antibiotic resistance, per year	3,326	3,070	–256
**Cost per patient, per year, €** ^†^			
Cost of PCT tests	0	87	87
Cost of antibiotic therapy	248	192	–56
AMR (due to difference in hospital LOS)	385	356	–30
Total cost difference	—	—	1.90
**Budget impact, per year, €** *			
Healthcare system	—	—	68,220
Per 1,000 patients	—	—	1,920

*Calculated using the total number of sepsis cases per year and the average duration of days patients spend on antibiotic therapy;

^†^Assuming a market share of 100%.

**Fig 2:**
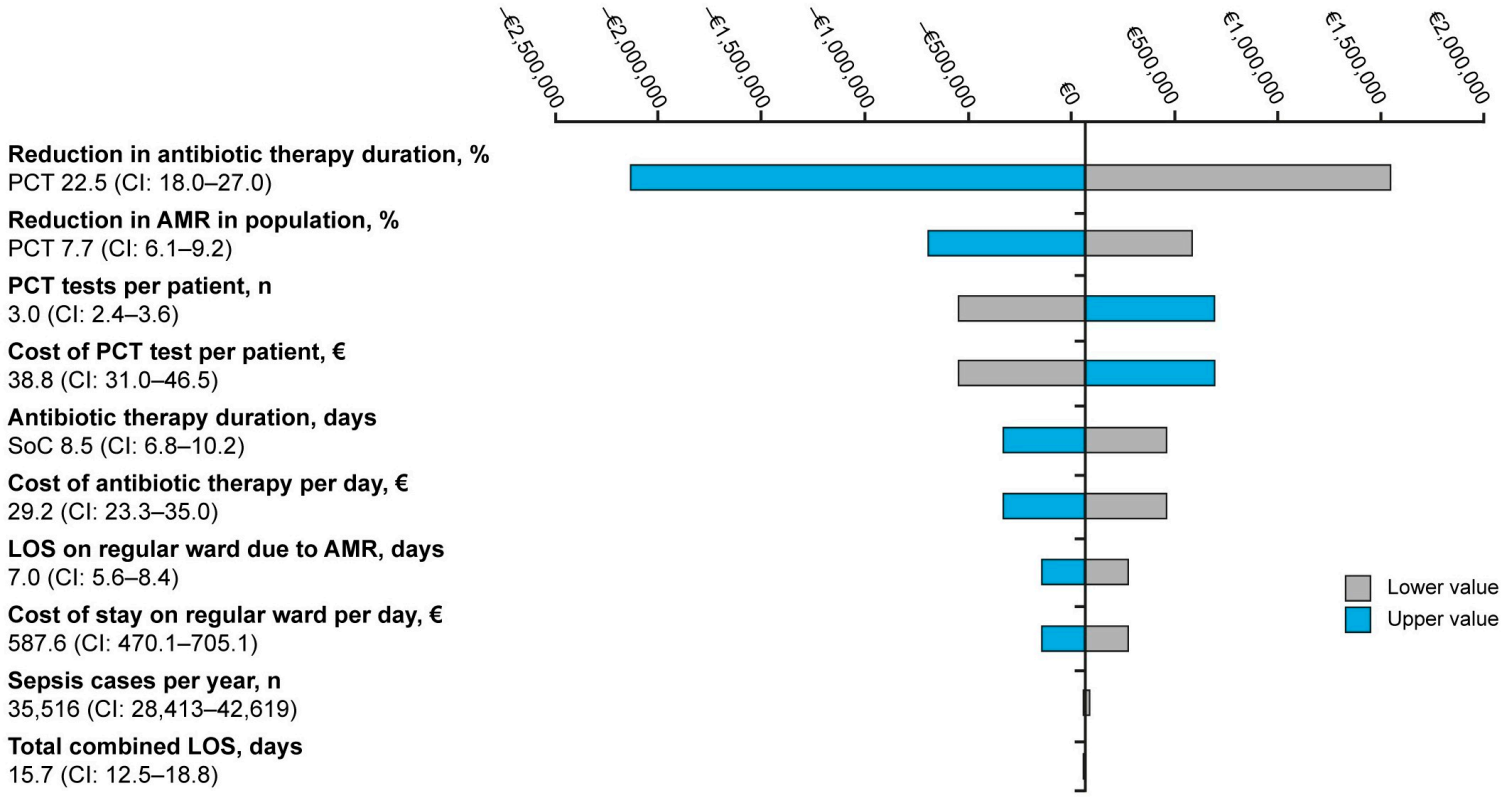
Tornado diagram–Impact of parameter variations, Belgium 2023. The tornado diagram illustrates the alteration from the base-case result if parameters are changed to the upper or lower value.

The ten drivers impacting the sensitivity analysis (Fig 2) are reduction in antibiotic therapy duration, reduction in AMR in population, number of PCT tests per patient, cost of PCT tests, antibiotic therapy duration, cost of antibiotic therapy, LOS on regular ward due to AMR, cost of stay on regular ward, sepsis cases, and total combined LOS. The sensitivity analysis shows great uncertainty, as the top eight drivers of the results are all able to change the outcome to be cost saving when changed to their upper or lower value of variability (Fig 2). Particularly the parameter for reduction in antibiotic therapy duration is shown to have a considerable effect on the results when compared to the other drivers.

### Scenario analysis

The scenario analysis considers the potential impact of PCT on LOS in the ICU and mechanical ventilation duration. The results indicate that a reduction of 1.46 days (13.9%; range: 11.1–16.6%) per patient in the ICU due to PCT testing would save the Belgian healthcare system €45.80M per year. Furthermore, the model shows that the reduction in mechanical ventilation duration due to PCT testing may result in €4.20M savings. The budget impact per patient in this scenario analysis would reach €–1,405, thus suggesting that by implementing PCT as a tool for antibiotic stewardship, the Belgian healthcare system would save €49.90M per year (Table 4).

**Table 4:** Scenario analysis expected results. Healthcare impact.

	SoC	PCT	Difference
**Cost per patient, per year, €** *			
Cost of hospital stay in the ICU	12,298	11,010	–1,288
Cost of mechanical ventilation	525	406	–119
Base-case analysis	—	—	2
Total cost difference	—	—	–1,405
**Budget impact, per year, million €** *			
Healthcare system	—	—	–49.90
Per 1000 patients	—	—	–1.41

*Assuming a market share of 100%.

**Fig 3:**
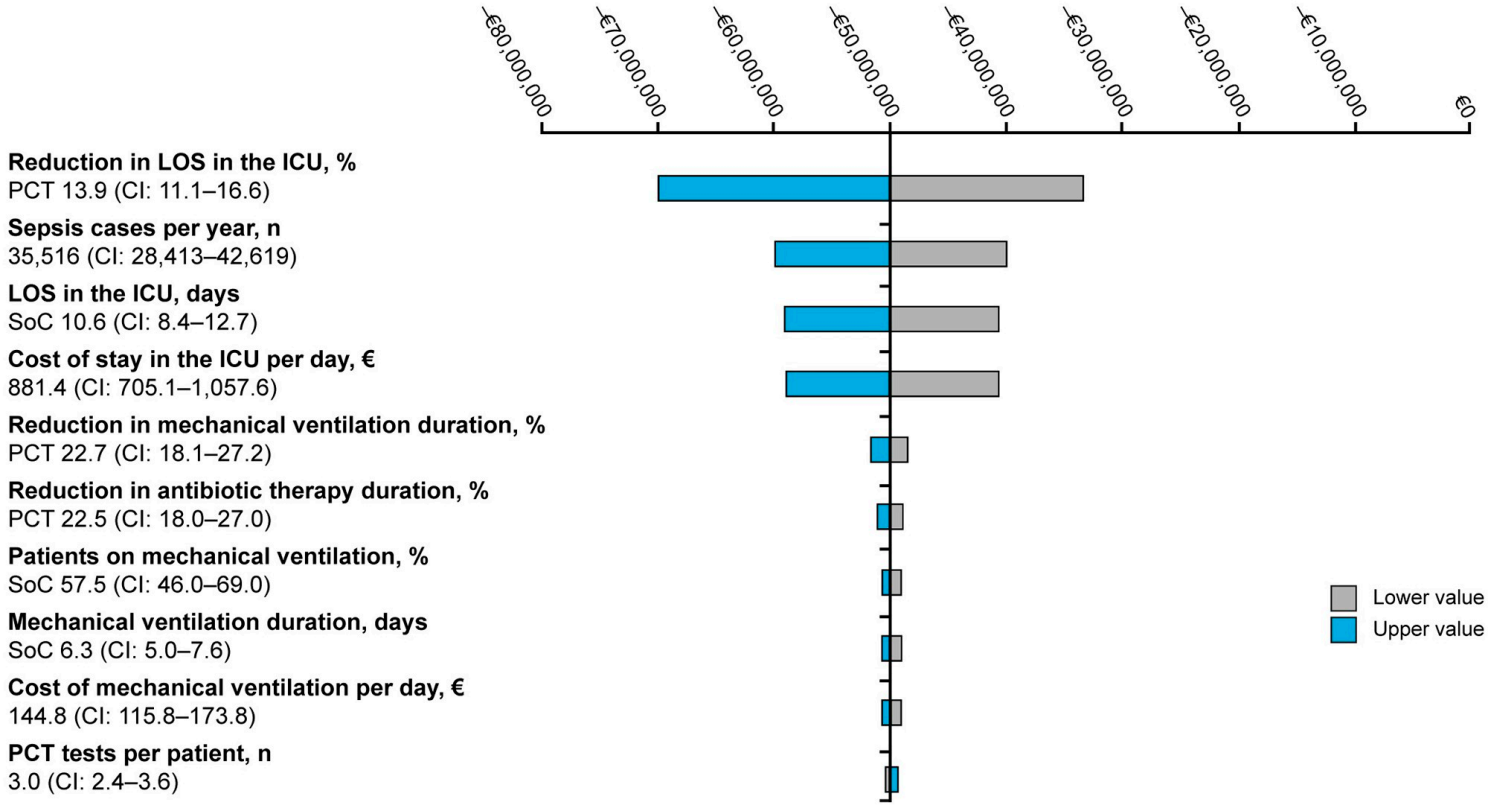
Scenario analysis, tornado diagram–Impact of parameter variations, Belgium 2023. The tornado diagram illustrates the alteration from the base-case result if parameters were changed to the upper or lower value.

The deterministic sensitivity analysis (Fig 3), of the scenario analysis, illustrates the key drivers of the results. The reduction in the LOS in the ICU is the main driver. Other key drivers are cases of sepsis per year, LOS in the ICU with SoC, and cost per ICU stay. In general, the model-based results were considered robust as varying the top ten ranked parameters to their upper or lower values still achieved cost savings.

## Discussion

AMR is an emerging health issue associated with potentially life-threatening consequences and substantial clinical and financial burden [4]. In the Belgian university hospitals, 30.5% of all patients are treated with antibiotics, and 52.7% of patients in the ICU receive antibiotic treatment [34]. Consequently, there is a need for new and improved patient management and the PCT assay could be a possible solution.

The objective of this model-based analysis was to analyze the budget impact of PCT as a tool for antibiotic stewardship compared to SoC for patients with suspected sepsis. To the best of our knowledge, this is the first budget impact analysis for this comparison in Belgium. The results indicated clinical benefits associated with PCT, such as a reduced exposure to antibiotics and a reduction in AMR infections. The results of the base-case analysis also indicated that a budget investment of €1.90 per patient is needed to implement PCT for patients with sepsis in the ICU. The Belgian healthcare system would need to invest €68,220 per year to implement PCT testing for patients with sepsis in the ICU. The scenario analysis predicted large budget savings associated with PCT, considering parameters such as reduction in LOS in the ICU and mechanical ventilation duration. When including the additional parameters, the Belgian healthcare system could potentially save up to €49.90M each year by following PCT-guided antibiotic treatment management. The base-case results were not robust based on the sensitivity analysis due to the influence of the key drivers on the outcome. For the scenario analysis, the results were found to be robust, as none of the drivers of the results altered the outcome.

There is a difference in the cost investment per patient from the base-case analysis to the study by Garay et al, of €1.90 versus $–101, respectively. One reason for this difference in budget impact is that this study did not assess the impact of PCT on *Clostridium difficile* (*C*. *diff*) [21]. Furthermore, the main reason that PCT does not generate cost savings in the base-case for Belgium, compared with the study from Argentina, is that the cost of antibiotic treatment is more than six times higher in Argentina compared to Belgium [21]. Therefore, a small change in antibiotic therapy duration has a larger impact on the result in Argentina compared with Belgium. When the scenario analysis is compared with the study by Mewes et al, €–1,405 versus $–11,311 respectively, a large difference between the two budget impact results can be explained by the cost of the ICU in the US being more than double that of Belgium. Furthermore, the reduction in the LOS in the ICU is also double in the US study compared to this study [22]. Other reasons include the differences in input parameters, as this study does not consider *C*. *diff* infections, reduction in laboratory tests performed or productivity losses.

The reason for not including additional parameters in the analysis is, by suggestion of clinical experts, that PCT should be considered an addition to the SoC for patients with suspected sepsis in Belgium, as PCT adds value over the use of C-reactive protein tests. Lastly, productivity loss is not in the scope of a healthcare system perspective in Belgium.

One important limitation of this study was the lack of Belgian specific data on PCT testing and pricing, mainly due to the fact that the tests are not reimbursed. Consequently, published international literature was used as input parameters in the health economic model. Another limitation is the use of a fixed range of variation (±20%), as this does not reflect the true variabilities.

## Conclusions

By implementing the PCT-guided algorithm for patients with suspected sepsis in the ICU, healthcare benefits are expected to be generated, such as reduced antibiotic exposure, reduced AMR infections, which could help to prolong the effectiveness of antimicrobial treatments globally. Predicted by the decision analytic model, the improved treatment management for sepsis comes with a healthcare investment of €1.90 per patient. However, when considering the potential reduction in the LOS in the ICU and mechanical ventilation duration, savings of €1,405 per patient are expected. In conclusion, the implementation of PCT tests may add clinical benefit to patients with sepsis, and generate considerable savings in the Belgian healthcare system.
